# The molecular species responsible for α_1_‐antitrypsin deficiency are suppressed by a small molecule chaperone

**DOI:** 10.1111/febs.15597

**Published:** 2020-11-11

**Authors:** Riccardo Ronzoni, Nina Heyer‐Chauhan, Annamaria Fra, Andrew C. Pearce, Martin Rüdiger, Elena Miranda, James A. Irving, David A. Lomas

**Affiliations:** ^1^ UCL Respiratory Division of Medicine University College London UK; ^2^ Department of Molecular and Translational Medicine University of Brescia Italy; ^3^ GSK Medicines Research Centre Stevenage UK; ^4^ Department of Biology and Biotechnologies ‘Charles Darwin’ and Pasteur Institute – Cenci‐Bolognetti Foundation Sapienza University of Rome Italy

**Keywords:** folding intermediate, polymer inhibitor, polymerisation, secretion, α_1_‐antitrypsin

## Abstract

The formation of ordered Z (Glu342Lys) α_1_‐antitrypsin polymers in hepatocytes is central to liver disease in α_1_‐antitrypsin deficiency. *In vitro* experiments have identified an intermediate conformational state (M*) that precedes polymer formation, but this has yet to be identified *in vivo*. Moreover, the mechanism of polymer formation and their fate in cells have been incompletely characterised. We have used cell models of disease in conjunction with conformation‐selective monoclonal antibodies and a small molecule inhibitor of polymerisation to define the dynamics of polymer formation, accumulation and secretion. Pulse‐chase experiments demonstrate that Z α_1_‐antitrypsin accumulates as short‐chain polymers that partition with soluble cellular components and are partially secreted by cells. These precede the formation of larger, insoluble polymers with a longer half‐life (10.9 ± 1.7 h and 20.9 ± 7.4 h for soluble and insoluble polymers, respectively). The M* intermediate (or a by‐product thereof) was identified in the cells by a conformation‐specific monoclonal antibody. This was completely abrogated by treatment with the small molecule, which also blocked the formation of intracellular polymers. These data allow us to conclude that the M* conformation is central to polymerisation of Z α_1_‐antitrypsin *in vivo*; preventing its accumulation represents a tractable approach for pharmacological treatment of this condition; polymers are partially secreted; and polymers exist as two distinct populations in cells whose different dynamics have likely consequences for the aetiology of the disease.

## Graphical abstract

The formation of ordered Z α_1_‐antitrypsin polymers is central to liver disease in α_1_‐antitrypsin deficiency. The nascent α_1_‐antitrypsin folds via the M* intermediate to native monomer or becomes incorporated into a soluble polymer that can be secreted, degraded or precipitate as insoluble inclusions. We describe the kinetics of the clearance of Z α_1_‐antitrypsin polymers and show that they can be abolished with a small molecule preventing the formation of M*.

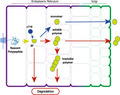

**Abbreviations**
BafA1: bafilomycin A1
ConA: concanavalin A
Endo H: endoglycosidase H
ER: endoplasmic reticulum
IP: immunoprecipitation
iPSCs: induced pluripotent stem cells
mAb: monoclonal antibodies
NP‐40: Nonidet P40
PAS: periodic acid–Schiff
PNGase F: peptide‐*N*‐glycosidase F
Tet‐On: tetracycline‐controlled transcriptional activation

## Introduction

α_1_‐Antitrypsin is the archetypal member of the serpin superfamily of proteins and a major inhibitor of neutrophil elastase in humans [1]. Plasma deficiency of this protease inhibitor predisposes to early‐onset panlobular basal emphysema due to deregulated elastase activity within the lung [2]. The common severe Z deficiency variant of α_1_‐antitrypsin (Glu342Lys) results in the formation of long chains of ordered aggregates termed polymers. These polymers accumulate within the endoplasmic reticulum (ER) of hepatocytes, condensing as periodic acid–Schiff (PAS)‐positive inclusions [1, 2, 3, 4, 5] that are associated with neonatal hepatitis, cirrhosis and hepatocellular carcinoma [6].

*In vitro* studies demonstrated that the Glu342Lys substitution perturbs folding of Z α_1_‐antitrypsin allowing the formation of a monomeric unstable intermediate, denoted M* [7]. This state is associated with changes in β‐sheet A and helix F [3, 7, 8, 9, 10] and is a precursor to oligomerisation involving an intermolecular domain swap [3, 11, 12]. Serpin polymerisation is a form of nonamyloid aggregation. The process involves β‐sheet interactions but is distinguished by an intermediate that remains largely well‐folded [13] with the subunits of the resulting polymers exhibiting only minimal structural perturbation with respect to the native conformation [11, 12, 14, 15, 16]. These ordered structures most likely explain the failure of polymers to activate the unfolded protein response in cellular and animal models of the disease [17, 18]. However, the accumulation of α_1_‐antitrypsin polymers within hepatocytes results in an increase in ER volume, increased intraluminal viscosity [19] and the formation of ER‐derived membrane‐bound inclusions [20]. Moreover, the extent of accumulation of Z α_1_‐antitrypsin and the associated hepatotoxicity depends on the efficiency of both ER‐associated degradation (ERAD) [21, 22, 23, 24] and lysosomal‐associated degradative pathways [25, 26, 27].

The structural variability of α_1_‐antitrypsin has resulted in the development of a toolkit of conformation‐specific monoclonal antibodies (mAb) that are able to recognise different conformations of this protein. The polymer‐specific 2C1 mAb [28, 29] that recognises intrahepatic α_1_‐antitrypsin polymers revealed that polymers of α_1_‐antitrypsin are present in the circulation of all individuals with Z α_1_‐antitrypsin deficiency [30]. The origin of these polymers is unclear, but our recent data from cell models of disease demonstrated indirectly that polymers can be secreted by cells [31]. The 5E3 mAb was used to characterise the polymerisation‐prone intermediate M* [32] *in vitro*, but has not been evaluated in a cellular model of α_1_‐antitrypsin deficiency.

We have used metabolic labelling and antibodies with different conformational preference (Table 1) to characterise the kinetics of polymer formation and resolution and the intracellular distribution and secretion of Z α_1_‐antitrypsin polymers in cellular models of disease.

**Table 1:** mAbs specificity.

*mAb*	*mAb specificity*	References
2C1	α1‐antitrypsin polymers	Miranda *et al*. [28]
3C11	Total α1‐antitrypsin	Tan *et al*. [30]
5E3	α1‐antitrypsin folding intermediate (M*)	Irving *et al*. [32]

## Results

### Intracellular Z α_1_‐antitrypsin polymers partition in both the NP‐40‐soluble and NP‐40‐insoluble fractions

**Fig. 1:**
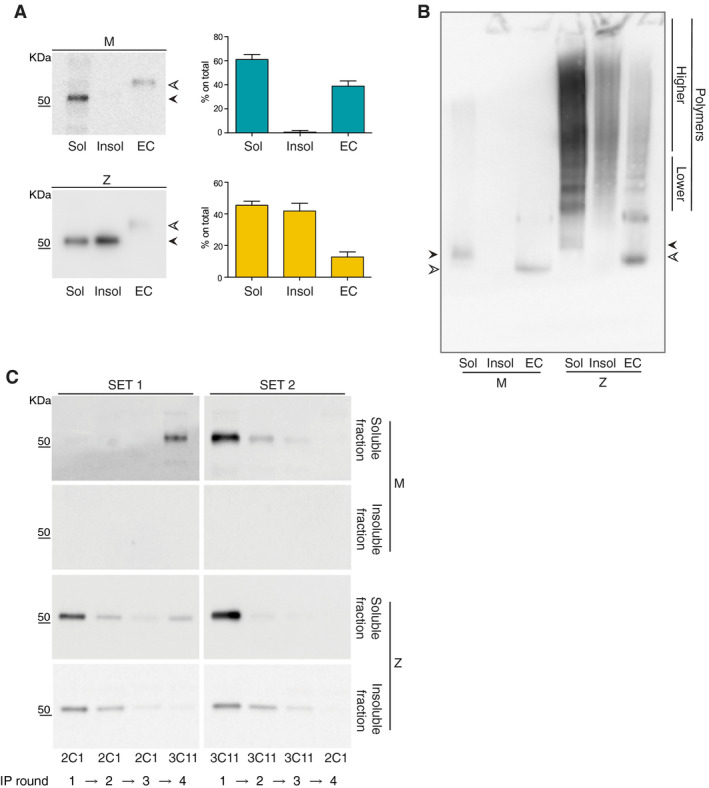
Identification of polymers in the soluble and insoluble intracellular fractions of cells expressing Z α_1_‐antitrypsin. (A) CHO‐inducible cells expressing either M or Z α_1_‐antitrypsin were induced with 0.5 μg·mL^−1^ doxycycline for 48 h and then lysed in 1% v/v NP‐40 buffer. The soluble (Sol), insoluble (Insol) and extracellular (EC) fractions were separated by 4–12% w/v acrylamide SDS/PAGE and the proteins visualised by immunoblotting for total α_1_‐antitrypsin with the 3C11 mAb. White and black arrowheads indicate the mature and immature glycosylated forms of α_1_‐antitrypsin, respectively. The percentage of intracellular and secreted α_1_‐antitrypsin was calculated by densitometric quantification. Graph shows mean ± standard error of the mean (± SEM, *n* = 3). (B) Cell lysates from CHO cells expressing either M or Z α_1_‐antitrypsin (as indicated) were concentrated with ConA beads and eluted as described in Methods. The samples were then resolved on a 3–12% w/v acrylamide nondenaturing gel followed by immunoblotting for total α_1_‐antitrypsin. White and black arrowheads indicate the mature and immature glycosylated forms of α_1_‐antitrypsin, respectively. The different charges acquired from the glycosylation process make the mature form of the protein run faster than the immature form. Monomeric secreted Z α_1_‐antitrypsin in the supernatant migrates more slowly in nondenaturing PAGE than secreted M α_1_‐antitrypsin as a result of the Glu342Lys mutation. The immunoblot is representative of 3 independent experiments. (C) 1% v/v NP‐40‐soluble and NP‐40‐insoluble fractions from cells expressing either M or Z α_1_‐antitrypsin were sequentially immunoprecipitated using the 2C1 polymer‐specific mAb (three times) and the 3C11 mAb against total α_1_‐antitrypsin (once) as indicated for Set 1. The fractions were also immunoprecipitated three times with the 3C11 mAb and once with 2C1 mAb as shown in Set 2. Proteins were eluted with an SDS‐based buffer and analysed by 4–12% w/v acrylamide SDS/PAGE followed by immunoblotting for α_1_‐antitrypsin with polyclonal antibody. Western blots are representative of two independent experiments.

A previously established and validated inducible tetracycline‐controlled transcriptional activation (Tet‐On) cellular system [19] was used to investigate the partitioning of intracellular polymers of Z α_1_‐antitrypsin. Chinese hamster ovary (CHO) K1 cells expressing Z or wild‐type M α_1_‐antitrypsin were induced with 0.5 μg·mL^−1^ doxycycline for 48 h and lysed in a buffer containing 1% v/v Nonidet P40 (NP‐40). Centrifugation at 12 000 ***g*** was used to separate the soluble from the insoluble fraction, and the latter was then mechanically resuspended in lysis buffer and sonicated. Biochemical analysis of these fractions and the culture medium supernatants by denaturing SDS/PAGE and immunoblot confirmed that M α_1_‐antitrypsin was only present in the cellular soluble and secreted fractions (Fig. 1A, upper panel and graph); the wild‐type variant is known to efficiently transit through the secretory pathway to the extracellular medium [19, 33, 34]. In contrast, Z α_1_‐antitrypsin accumulated in both the NP‐40‐soluble and NP‐40‐insoluble fractions and was present at lower level in the cell medium compared with M α_1_‐antitrypsin (Fig. 1A, lower panel and graph).

The NP‐40‐soluble and NP‐40‐insoluble fractions were then analysed by nondenaturing PAGE and immunoblot for total α_1_‐antitrypsin (Fig. 1B). The soluble fraction obtained from cells expressing M α_1_‐antitrypsin migrated as a single monomeric band, while the soluble fraction from cells expressing Z α_1_‐antitrypsin showed a ladder of higher molecular weight forms consistent with a population of α_1_‐antitrypsin polymers of variable size [4]. Of note, the broad profile of the soluble fraction exhibited an overall faster migration (Fig. 1B, lower) and contained distinct bands that matched, allowing for differences in *N*‐glycosylation maturation states, with corresponding bands in the supernatant. The insoluble fraction, instead, was characterised by a high molecular weight‐shifted smear and the absence of low molecular weight bands. A fraction of this sample was trapped in the stacking gel suggesting the additional presence of larger, higher molecular weight species.

The presence of α_1_‐antitrypsin monomers and polymers in the soluble and insoluble intracellular fractions was analysed by sequential immunoprecipitation (IP) using antibodies with differential conformational selectivity (Table 1 and Fig. 1C). First, polymers were immunoprecipitated three times with the antipolymer 2C1 mAb [28] and then residual monomer captured in one round with the non‐conformation‐selective 3C11 mAb [30], using the supernatant of each IP as the input for the next one (Fig. 1C, *SET 1*). Cells expressing M α_1_‐antitrypsin showed a single band positive for 3C11 in the soluble fraction and no 2C1 recognition in either cellular fraction, confirming its monomeric state under the expression conditions used. The soluble fraction from cells expressing Z α_1_‐antitrypsin was positive for 2C1, and Z α_1_‐antitrypsin could still be immunoprecipitated with 3C11 after three rounds of polymer depletion. In contrast, three rounds of IP of the insoluble fraction with 2C1 depleted virtually all α_1_‐antitrypsin, with no residual nonpolymeric material recognised by the 3C11 mAb. In a control experiment, the order of the mAbs was reversed (three rounds with 3C11 and a final one with 2C1). As expected, all α_1_‐antitrypsin was immunoprecipitated by the non‐conformation‐selective 3C11 mAb (Fig. 1C, *SET 2*).

### Z α_1_‐antitrypsin polymers are secreted through the canonical secretory pathway

**Fig. 2:**
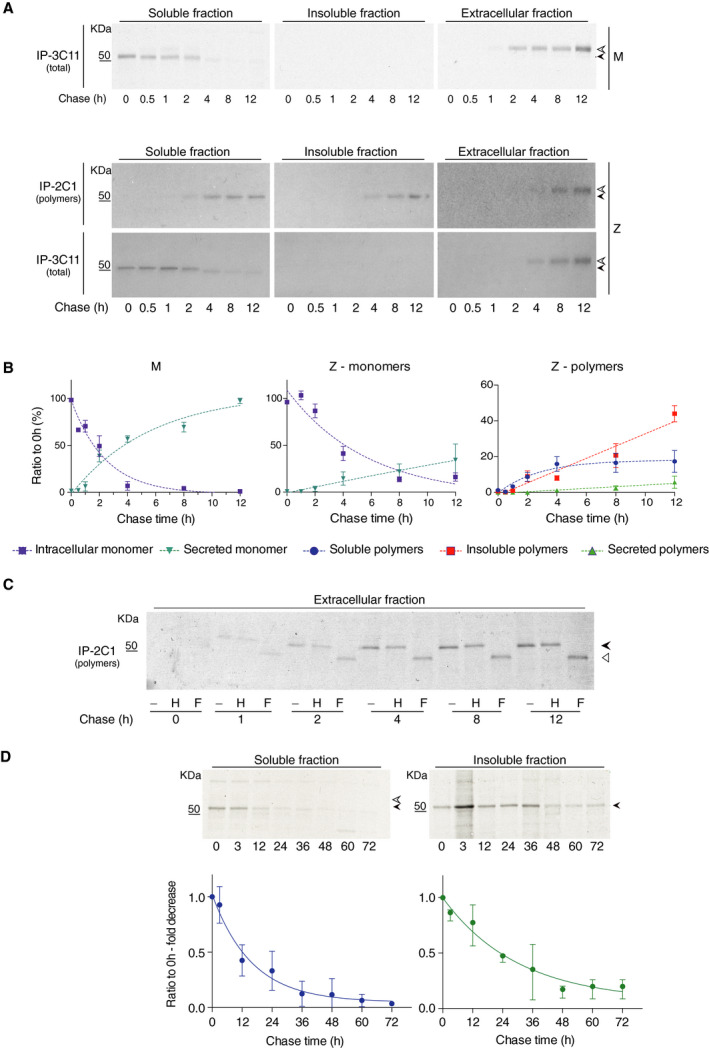
Kinetics of intracellular accumulation and secretion of Z α_1_‐antitrypsin polymers. (A) CHO‐K1 Tet‐On cells expressing either M or Z α_1_‐antitrypsin were induced with doxycycline for 48 h. Cells were labelled for 10 min with ^35^S Met/Cys and chased at the indicated times. Culture media were collected and cells lysed in 1% v/v NP‐40 buffer. Intracellular fractions and culture media from cells expressing Z α_1_‐antitrypsin were immunoprecipitated twice with the polymer‐specific mAb (2C1) and then with a mAb against total α_1_‐antitrypsin (3C11). Samples were resolved by 4–12% w/v acrylamide SDS/PAGE and detected by autoradiography. White and black arrowheads indicate the mature and immature glycosylated forms of α_1_‐antitrypsin, respectively. Autoradiograms are representative of three independent experiments. (B) Densitometric analysis of the bands in Fig. 2A. Graph shows mean ± standard error of the mean (± SEM, *n* = 3). Densitometry of the bands was analysed by image studio software. Dotted lines are only intended to indicate the trend of the data. (C) The media from the cell culture expressing Z α_1_‐antitrypsin, shown in panel A, were treated with either endo H (H) or PNGase F (F) or not treated (−). Samples were then analysed by SDS/PAGE and detected by autoradiography. Black and white arrowheads indicate the glycosylated and deglycosylated forms of α_1_‐antitrypsin, respectively. (D) CHO‐K1 Tet‐On cells expressing Z α_1_‐antitrypsin were induced with doxycycline for 48 h, labelled for 12 h with ^35^S Met/Cys and then incubated for 12 h with normal medium with 0.5 μg·mL^−1^ doxycycline. Culture media were collected at the specific chase time points and cells lysed in 1% v/v NP‐40 buffer. The intracellular fractions were immunoprecipitated with the 2C1 polymer‐specific mAb, and samples were separated on a 4–12% w/v acrylamide SDS/PAGE and detected by autoradiography. Graph shows mean ± standard deviation (soluble polymers ± SD, *n* = 7, insoluble polymers ± SD, *n* = 2). Densitometry of the bands was analysed by image studio software. The gels are representative of the experiments reported in Table 2.

Radiolabeling‐based approaches provide a means to follow the molecular fate of a protein in a dynamic system without the perturbations that may be introduced by fluorescent tags. We investigated the kinetics of formation and accumulation of α_1_‐antitrypsin polymers by pulse‐chase experiments. CHO K1 cells expressing M or Z α_1_‐antitrypsin were pulse‐labelled for 10 min with ^35^S‐methionine and cysteine, and at the chase times indicated in Fig. 2A, the culture medium was collected and the cells lysed in NP‐40 buffer. M α_1_‐antitrypsin samples were immunoprecipitated with 3C11 mAb to recover all α_1_‐antitrypsin (monomeric, as demonstrated in Figs 1 and 2A, top panels, and Fig. 2B, left panel), while the Z α_1_‐antitrypsin samples were sequentially immunoprecipitated with the 2C1 antipolymer mAb (Fig. 2A, middle panels and Fig. 2B, Z‐polymers) and then with the 3C11 mAb (Fig. 2A, lower panels and Fig. 2B, Z‐monomers).

As shown in Fig. 2A,B, in cells expressing the Z variant, radiolabelled monomeric α_1_‐antitrypsin was detectable at the initial 0 h time point, but intracellular soluble polymers only became visible after a delay of 2 h and gradually increased to plateau at 8 h. Soluble extracellular polymers became detectable after 4 h of chase. In the insoluble fraction, polymers were apparent from the 8‐h time point and also increased with time, without exhibiting a plateau over the duration of the experiment.

In the autoradiographs (Fig. 2A), the secreted material (both monomeric and polymeric Z α_1_‐antitrypsin, white arrowheads) had a higher molecular mass compared with the intracellular species (black arrowheads), as is characteristic of mature glycoproteins secreted through the canonical secretory pathway [28, 31]. Analysis of the *N*‐linked glycosylation state was performed to assess the type of glycans present in the extracellular polymers. Polymers immunoprecipitated by 2C1 mAb from culture media of Z α_1_‐antitrypsin cells pulsed as in Fig. 2A were subjected to digestion with peptide‐*N*‐glycosidase F (PNGase F), which removes all types of *N*‐linked glycans, or endoglycosidase H (endo H), which cleaves pre‐Golgi glycans only. All secreted α_1_‐antitrypsin was sensitive to digestion by PNGase F but resistant to treatment with endo H (Fig. 2C). This demonstrated that at all time points only mature glycans were present in extracellular polymers and therefore that their presence in the media was the result of passage through the secretory pathway and not a cell lysis‐related artefact.

### Insoluble Z α_1_‐antitrypsin polymers have a longer intracellular half‐life than soluble polymers

Soluble Z α_1_‐antitrypsin polymers reached a peak at approximately 4 h and remained stable until 12 h, while insoluble polymers were still increasing after 12 h of chase (Fig. 2A). The kinetics of clearance of these polymers was assessed with a long metabolic labelling experiment. CHO K1 cells expressing Z α_1_‐antitrypsin were metabolically labelled with ^35^S methionine and cysteine for 24 h. At the end of the labelling time, the radioactive culture medium was replaced with normal medium and the cells were grown for a further 12 h. This prolonged ‘conversion time’ was designed to promote the incorporation of all the radioactive monomeric Z α_1_‐antitrypsin molecules into polymers. Following the conversion period, cells were chased for up to 72 h and polymeric α_1_‐antitrypsin was immunoprecipitated with the 2C1 mAb (Fig. 2D). This analysis demonstrated that soluble polymers were cleared more rapidly from cells than the insoluble counterpart. The half‐time for the clearance of intracellular soluble polymers was 10.9 ± 1.7 h, while the half‐time for the insoluble polymers was 20.9 ± 7.4 h (Table 2). It is notable that while soluble polymers were almost entirely cleared following the 72‐h chase, a residual insoluble component remained.

**Table 2:** Soluble and insoluble polymer half‐life.

		Half‐life (h)	SE	No. of indep. exps.	*df*
CHO K1 AAT Z	Soluble polymers	10.9	1.7	7	47
	Insoluble polymers	20.9	7.4	2	13
iPSC‐derived hepatocytes	Soluble polymers	14.7	6.2	5	29
	Insoluble polymers	19.1	4.9	2	9

In order to verify the data obtained in the inducible CHO K1 cell lines and to expand our analysis to a more physiologically relevant context, the same approach was applied to induced pluripotent stem cell (iPSC)‐derived hepatocytes. These had been generated previously using fibroblasts from an individual homozygous for the Z variant of α_1_‐antitrypsin [35, 36]. iPSC‐derived hepatocytes predifferentiated for 23 days were revived from frozen stocks and kept in specific medium and under hypoxic conditions in order to promote further hepatic differentiation [37]. At day 35 postdifferentiation, cells were starved, radioactively labelled with ^35^S methionine and cysteine for 24 h and incubated for 12 h in nonradioactive medium to allow the conversion of radioactive α_1_‐antitrypsin monomers into polymers. Culture media were collected and cells processed every 12 h up to 108 h postpulse. Intracellular soluble and insoluble fractions of α_1_‐antitrypsin polymers were immunoprecipitated with the 2C1 mAb and analysed as above. In this cell system, soluble polymers also exhibited a shorter half‐time 14.6 ± 6.2 h compared with 19.1 ± 4.9 h for insoluble polymers (Table 2).

### Circulating polymers of Z α_1_‐antitrypsin have a predominantly intracellular origin

**Fig. 3:**
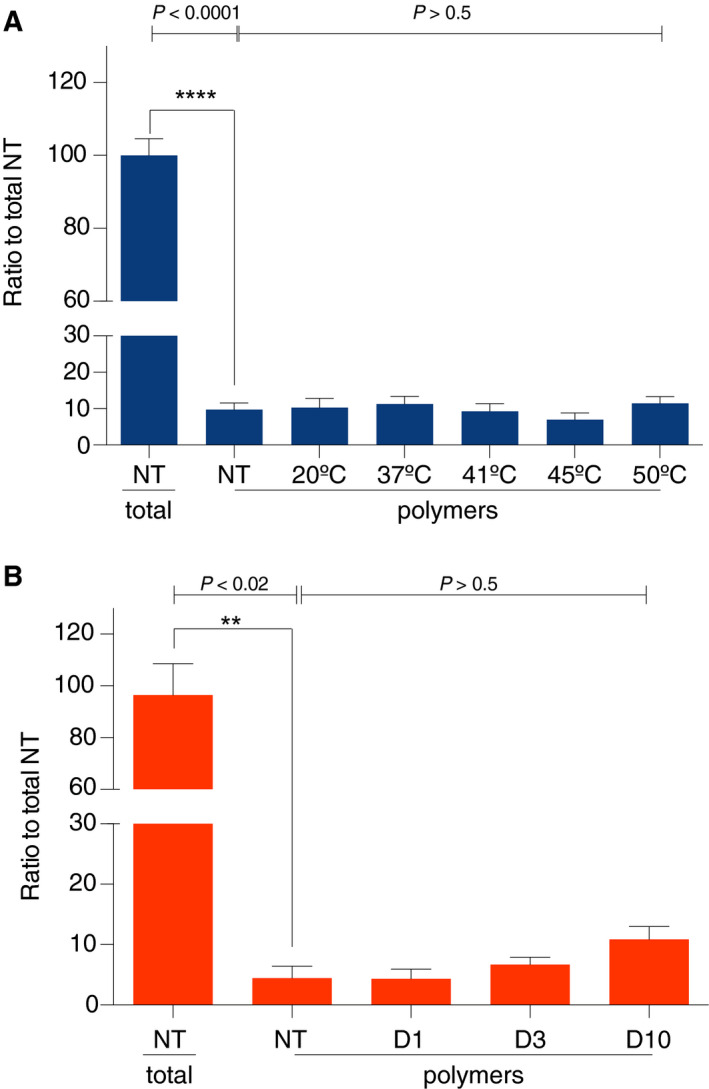
Extracellular polymers are not formed post‐secretion. (A) Plasma samples from three different Pi ZZ α_1_‐antitrypsin individuals were heated for 24 h at 20, 37, 41, 45 and 50 ºC and subsequently analysed by sandwich ELISA, using the 3C11 mAb (total α_1_‐antitrypsin) or 2C1 mAb (α_1_‐antitrypsin polymers) as capture antibodies and a polyclonal antibody HRP‐conjugated for detection. The graphs show mean ± standard error of the amount estimated relative to the respective standard curves (± SEM, *n* = 3; one‐way ANOVA, Bonferroni's multiple comparisons test, *df* = 34). (B) The same plasma samples were incubated at 37 ºC for 1, 3 and 10 days, and subsequently analysed by the same sandwich ELISA. The graphs show mean ± standard error (± SEM, *n* = 3; one‐way ANOVA, Bonferroni's multiple comparisons test, *df* = 8). Statistical analyses were performed using graphpad prism.

Our data provide direct evidence that polymers are actively secreted by cells. This has potential implications for the interpretation of polymers identified in patient plasma [30]. However, while plasma contains a complex mixture of molecules, including sugars and proteins such as serum albumin which are known to act as stabilisers against denaturation [38], it is unknown whether polymers can form spontaneously in the circulation. A previous experiment in which plasma samples from Z α_1_‐antitrypsin homozygotes were incubated *in vitro* at 37 °C for 3 days showed no increase in polymer content [30]. To extend this study, plasma samples from three different Z α_1_‐antitrypsin homozygote individuals were incubated at 20, 37, 41, 45 and 50 °C for 24 h and polymer formation was assessed by ELISA with the 2C1 mAb relative to recognition of the total α_1_‐antitrypsin population by the 3C11 mAb (Fig. 3A, first bar). There was no significant increase in the α_1_‐antitrypsin polymer signal following 24‐h incubation over this range of temperatures (Fig. 3A, bars 3 to 7). Plasma samples from the same patients were also incubated at 37 °C for 1, 3 and 10 days, representing 0.2‐, 0.6‐ and twofold the half‐life of Z α_1_‐antitrypsin in the circulation [39], and analysed again by 2C1 mAb ELISA. Again, no significant difference in polymer signal was observed after 3 and 10 days of incubation (Fig. 3B).

The plasma samples were also concentrated by concanavalin A (ConA) pull‐down and analysed on a nondenaturing gel followed by western blot with the 2C1 mAb (Fig. S1). This analysis showed a small but nonsignificant increase in polymer content after 10 days. Together, these data support the conclusion that Z α_1_‐antitrypsin has marked stability in plasma and accordingly the primary source of circulating polymer is most likely secretion from cells.

### Soluble and insoluble polymers are cleared by different mechanisms of degradation

**Fig. 4:**
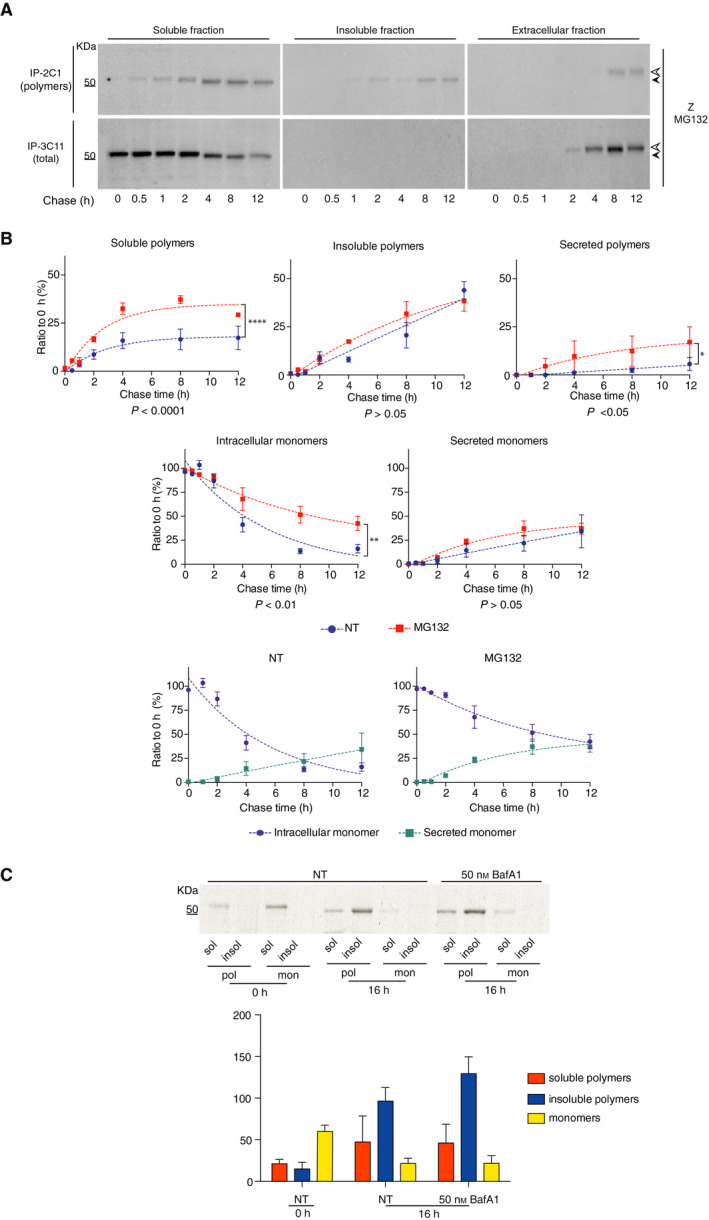
Inhibition of the proteasome increases soluble intracellular Z α_1_‐antitrypsin polymers. (A) CHO‐K1 Tet‐On cells expressing Z α_1_‐antitrypsin were induced with doxycycline for 48 h, treated with 80 µm MG132, labelled for 10 min with ^35^S Met/Cys and chased at the reported times. Culture media were collected and cells lysed in 1% v/v NP‐40 buffer. Intracellular fractions and culture media were immunoprecipitated twice with the polymer‐specific mAb 2C1 and then with the 3C11 mAb against total α_1_‐antitrypsin. Samples were resolved by 4–12% w/v acrylamide SDS/PAGE and detected by autoradiography. White and black arrowheads indicate the mature and immature glycosylated forms of α_1_‐antitrypsin, respectively. Representative of three independent experiments. (B) Graphs show densitometric analysis of MG132 pulse‐chase experiments in (A) (mean ± SEM, *n* = 3). Densitometry of the bands was performed by image studio software. Statistical analysis (two‐way ANOVA) was calculated using graphpad prism. Dotted lines are intended to indicate the trend of the data only. Protein precipitated in the first round of IP with the 2C1 mAb is denoted ‘polymer’. ‘Monomer’ refers to residual α_1_‐antitrypsin immunoprecipitated by the 3C11 mAb after two rounds of IP with the 2C1 mAb. (C) CHO cells expressing Z α_1_‐antitrypsin were induced with 0.5 μg·mL^−1^ doxycycline and treated with either 50 nm BafA1 or 0.1% v/v DMSO (NT) for 16 h. After lysis at the indicated times, 1% v/v NP‐40‐soluble and NP‐40‐insoluble fractions were separated and sequentially immunoprecipitated with the 2C1 antipolymer mAb and once with the 3C11 mAb that detects all conformers of α_1_‐antitrypsin. The eluted samples were resolved on 4–12% w/v acrylamide SDS/PAGE followed by immunoblotting for total α_1_‐antitrypsin. The top panel is representative of three independent experiments, and the graph shows mean ± standard error of the mean (± SEM, *n* = 3). Densitometry of the bands was performed by image studio software. ‘Polymer’ denotes the protein precipitated in the first round of IP with the 2C1 mAb. ‘Monomer’ refers to residual α_1_‐antitrypsin immunoprecipitated by the 3C11 mAb after two rounds of IP with the 2C1 mAb.

Endoplasmic reticulum‐associated degradation, mediated by the proteasome, has previously been identified as the major contributor to turnover of the Z α_1_‐antitrypsin variant [9, 22, 40, 41, 42, 43]. CHO K1 cells expressing Z α_1_‐antitrypsin were subjected to pulse chase in the presence of the reversible inhibitor MG132 in order to investigate the effect of proteasomal degradation on the kinetics of accumulation and secretion of the polymer populations. Treatment with the inhibitor, compared with the kinetics of untreated cells shown in Fig. 2 (and reported in Fig. 4, lower panel, NT), resulted in increased accumulation of polymers in the soluble but not in the insoluble intracellular fractions, and increased polymeric and total α_1_‐antitrypsin in the culture medium (Fig. 4A, upper panel and Fig. 4B, upper panels). MG132 did not appreciably alter the accumulation of monomer in the supernatant over time, but instead led to the intracellular retention of a monomeric component (Fig. 4B, lower panels).

Recent work has shown that in addition to an autophagy‐mediated mechanism [43], Z α_1_‐antitrypsin polymers are degraded via a distinct lysosome‐associated system [27]. We thus evaluated the clearance of soluble and insoluble intracellular polymers by lysosomes in our cell model. CHO K1 cells expressing Z α_1_‐antitrypsin were induced with doxycycline for 48 h, labelled for 15 min with ^35^S methionine and cysteine and then treated for 16 h with either 50 nm bafilomycin A1 (BafA1), a drug capable of inhibiting the lytic activity of lysosomes, or with a DMSO control. Cells were processed at different time points as shown in Fig. 4C, and intracellular fractions were immunoprecipitated first with the 2C1 mAb for polymers and then with the 3C11 mAb for residual nonpolymeric α_1_‐antitrypsin. Treatment with BafA1 caused a small but nonsignificant increase in the quantity of insoluble polymers but had no effect on soluble polymers or monomeric Z α_1_‐antitrypsin.

### An experimental compound, c716, prevents intracellular polymer formation and increases secretion

**Fig. 5:**
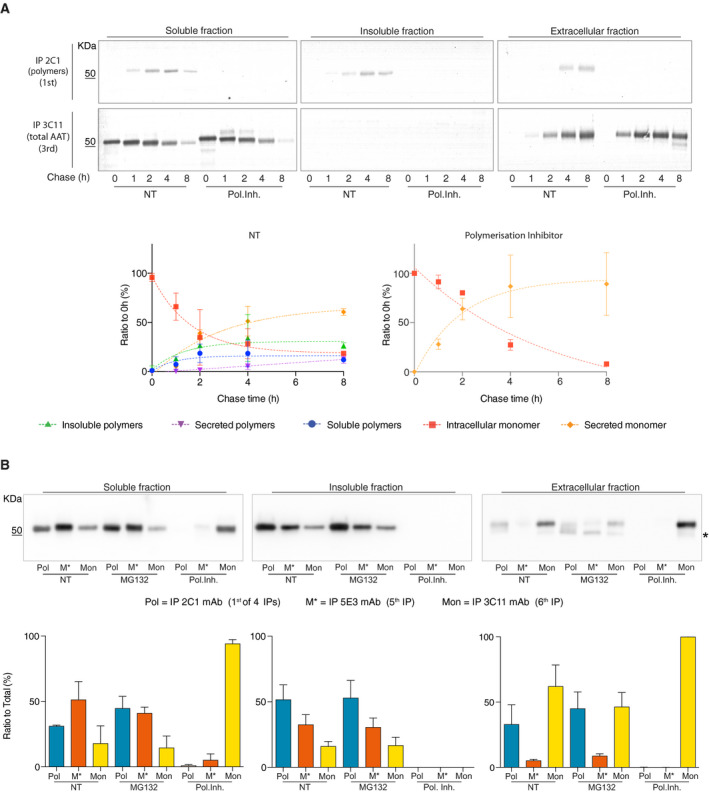
Detection of the folding intermediate M* in cells. (A) CHO‐K1 Tet‐On cells expressing Z α_1_‐antitrypsin were induced with doxycycline and treated either with the small molecule polymerisation inhibitor (c716) or with 0.1% v/v DMSO for 48 h and labelled for 10 min with ^35^S Met/Cys. Culture media were collected and cells lysed in 1% v/v NP‐40 buffer as described above. Intracellular fractions and cell media were immunoprecipitated with the 2C1 antipolymer mAb and then the 3C11 total α_1_‐antitrypsin mAb. Samples were resolved by 4–12% w/v acrylamide SDS/PAGE and the proteins detected by autoradiography. Densitometry of the bands was performed using image studio software. ‘Polymer’ indicates the protein precipitated in the first round of IP with the 2C1 mAb. ‘Monomer’ refers to residual α_1_‐antitrypsin immunoprecipitated by the 3C11 mAb after two rounds of IP with the 2C1 mAb. NT represents the densitometric analysis of untreated cells, while ‘polymer inhibitor’ refers to cells treated with the experimental small molecule (c716). The top panels are representative of two independent experiments, and the graphs show mean ± standard deviation (± SD, *n* = 2). Dotted lines do not show the fit but indicate the trend of the data. (B) CHO cells expressing Z α_1_‐antitrypsin were induced with 0.5 μg·mL^−1^ doxycycline and treated with either the small molecule inhibitor of polymerisation (c716) or 0.1% v/v DMSO for 48 h. Cells treated with proteasome inhibitor were incubated with 80 µm MG132 1 h before the beginning of the secretion assay. After washing and incubation in Opti‐MEM, the NP‐40‐soluble and extracellular fractions were separated and sequentially immunoprecipitated four times with the 2C1 antipolymer mAb, once with the 5E3 anti‐M* mAb and once with the 3C11 mAb that detects all conformers of α_1_‐antitrypsin. The eluted samples were resolved on 4–12% w/v acrylamide SDS/PAGE followed by immunoblotting with a rabbit polyclonal anti‐α_1_‐antitrypsin (only the first 2C1 IP is shown). The top panels are representative of two independent experiments, and the graphs show mean ± standard deviation (± SD, *n* = 2). Asterisk (*) indicates signals from intracellular material released by dead cells. Dotted lines do not show the fit but indicate the trend of the data.

We have developed a small molecule inhibitor of polymerisation, c716, that is active *in vivo* and that prevents the formation of M* *in vitro* [44]. To investigate its behaviour under the experimental conditions considered here, CHO K1 cells were seeded in the presence of DMSO or c716 and subsequently induced to produce Z α_1_‐antitrypsin for 48 h before being starved, pulsed and processed as described in Fig 1. Intra‐Z α_1_‐antitrypsin and extracellular Z α_1_‐antitrypsin were sequentially immunoprecipitated with 2C1 and 3C11 mAbs. The experimental compound abolished the formation of both soluble and insoluble polymers (Fig. 5A, upper panels and graphs) and increased the secretion of Z α_1_‐antitrypsin (Fig. 5A, lower right panel and graphs). Indeed, treatment with the compound resulted in rates of secretion similar to those of the wild‐type M α_1_‐antitrypsin (compare Fig. 5A, right graph with Fig. 2B, left graph), increasing the amount secreted after 1 h by ~ 5‐fold from 5.2 ± 0.2% (± SD, *n* = 2) to 28.0 ± 5.3 % (± SD, *n* = 2).

### The monomeric intermediate M* is the key species for Z α_1_‐antitrypsin intracellular polymer formation

*In vitro* experiments have shown that α_1_‐antitrypsin folds via an intermediate ensemble [43] before reaching its native monomeric state [13, 45, 46]. Folding is rapid for wild‐type M α_1_‐antitrypsin but is delayed for the Z mutant, increasing the population of these intermediate conformations [47]. One of these conformations, that we have termed M*, is a precursor to polymer formation and elongation [3, 7, 48, 49]. This molecular species, or a monomeric by‐product thereof, is sufficiently persistent to be detectable *in vitro* [11, 46, 49] and is recognised by the 5E3 mAb [32] (Table 1). The presence of M* in a cellular environment has not been established; we therefore made use of the 5E3 mAb and c716 to determine its role in intracellular polymer formation.

CHO K1 cells expressing Z α_1_‐antitrypsin were induced with doxycycline and treated for 8 h with DMSO, c716 or MG132. Intra‐ and extracellular fractions were collected and subjected to sequential IPs, first with the 2C1 antipolymer mAb (four rounds to guarantee complete depletion, Fig. S2), once with the anti‐M* 5E3 mAb and finally with the 3C11 antitotal α_1_‐antitrypsin mAb. 2C1‐positive polymers were efficiently immunoprecipitated from the NP‐40‐soluble intracellular fraction (Fig. 5B, left panels). However, a fraction remained that was immunoprecipitated by the 5E3 mAb and a residual amount of α_1_‐antitrypsin that was immunoprecipitated by the 3C11 mAb, corresponding to the native monomer. Inhibition of ERAD with the proteasomal inhibitor MG132 slightly increased the quantity of polymers recognised by 2C1 mAb with a corresponding decrease in monomers detected by the 3C11 mAb.

The extracellular fraction was characterised by the presence of polymers and native folded monomers (Fig. 5B, right panel, NT lanes); treatment with MG132 increased the secretion of α_1_‐antitrypsin polymers and decreased the accumulation of monomer (Fig. 5B, right panel, MG132 lanes). It is interesting to note that little extracellular material could be immunoprecipitated by 5E3 mAb, demonstrating the intracellular nature of this folding intermediate and the specificity of the mAb. Stabilisation of a native‐like state using c716 reduced the formation of M* and polymers, favouring the secretion of the monomeric form of Z α_1_‐antitrypsin (Fig. 5B, right panel). The level of insoluble polymers remained unchanged upon treatment with MG132, while treatment with c716 completely abolished the presence of polymers and M* in this fraction (Fig. 5A).

## Discussion

The Z (Glu342Lys) mutant of α_1_‐antitrypsin misfolds and forms linear unbranched polymer chains in the ER. It has been shown in cell models of disease that proteostatic degradative mechanisms – ERAD, autophagy and lysosomal‐associated degradation – compensate to some degree for this intracellular burden of misfolded and aggregated protein [18, 21, 22, 27, 42, 43]. However, a hallmark of α_1_‐antitrypsin deficiency is the deposition of polymers of mutant α_1_‐antitrypsin as PAS‐positive inclusions within hepatocytes [3]. The processes that result in this retention of α_1_‐antitrypsin are incompletely characterised.

Polymers of α_1_‐antitrypsin partition into components of cellular extracts that can be defined as ‘NP‐40‐soluble’ and ‘NP‐40‐insoluble’ [42, 50], but it has not been determined whether these reflect a single population of molecules or two populations with distinct characteristics. Here, we investigated in detail the kinetics of formation, deposition and secretion of Z α_1_‐antitrypsin polymers. Our data show that the two populations of polymers with differential solubility do indeed exhibit different characteristics: the NP‐40‐soluble fraction contained a mixture of monomers and lower molecular weight polymers, with a shorter cellular half‐life and whose levels increased in the presence of the proteasomal inhibitor MG132. In contrast, the NP‐40‐insoluble fraction lacked monomers, had a generally larger polymer size distribution, exhibited a longer half‐life and was unaffected by MG132. Notably, the latter population appeared to include a component that was not degraded by the cellular proteostatic mechanisms over the course of a 72 h of experiment. We speculate that this may represent the species that form intractable inclusions within the liver of Z α_1_‐antitrypsin individuals. A limited subset of polymers appeared in the culture medium at a similar rate to their appearance in the intracellular soluble fraction and with a similar size profile, suggesting progression from formation to secretion. These extracellular polymers were sensitive to PNGase F but resistant to endo H digestion, in keeping with the maturation of *N*‐glycans during transit through the Golgi complex along the canonical secretory pathway [51]. Additionally, the faster migration of the bands under electrophoresis of the soluble fraction from 4 h onwards is consistent with trimming of *N*‐glycans that occurs in the ER or in the cis‐Golgi [52].

These modifications were not seen in insoluble polymers, suggesting that they are inaccessible to the enzymes that process *N*‐glycans. Treatment with the proteasome inhibitor MG132 increased the formation of intracellular soluble and secreted polymers but had no effect on insoluble intracellular polymers. This supports the hypothesis that while soluble polymers are trafficked through the secretory pathway and into the extracellular space, insoluble polymers represent a molecular endpoint that cannot be resolved by proteasomal degradation but that must be resolved by alternative cellular homeostatic processes such as autophagy.

The CHO cell model faithfully recapitulates the handling of mutant variants of α_1_‐antitrypsin seen in other cellular models [23, 53], with the benefits of inducible and titratable expression and robust characteristics in cell culture. We confirmed our findings using an iPSC model of Z α_1_‐antitrypsin deficiency [35, 36], which showed similar half‐times for the clearance of soluble and insoluble polymers. In both cellular models, a longer turnover time for insoluble polymers, together with the differing effect of proteasomal inhibition on polymer pools, suggests that soluble and insoluble polymers are handled in different ways; smaller and more soluble polymers can be secreted, whereas larger ones, which we hypothesise represent the precursors of the inclusion bodies seen in pathological specimens, are more persistent and only partially degraded by an alternative pathway related to lysosomal degradation [43, 54].

We have shown previously that polymers of α_1_‐antitrypsin can be detected in the circulation of all subjects who carry a Z allele [55]. Our observation that polymers of Z α_1_‐antitrypsin can be secreted by cells *in vitro*, coupled with the inability to induce polymer formation in plasma samples incubated at either high temperature or for prolonged times, allows us to conclude that the polymers found in circulation are most likely the result of secretion of soluble polymers from hepatocytes. Circulating polymer levels therefore represent a potential ‘window’ into the efficacy of the proteostatic processes that regulate the formation and accumulation of α_1_‐antitrypsin polymers in the liver of individuals with α_1_‐antitrypsin deficiency.

Many *in vitro* studies have established that α_1_‐antitrypsin folds via an intermediate ensemble [13, 27, 32, 56] that samples a polymerisation‐prone intermediate state that we have termed M* [3, 7, 11, 13, 33, 47, 49]. However, the presence of M* within the ER as an intermediate on the Z α_1_‐antitrypsin secretion pathway has not been established. Using the 5E3 mAb that detects M* [43] and a small molecule that stabilises α_1_‐antitrypsin against M* formation, we identified three different conformational species within the NP‐40‐soluble fraction in cells expressing Z α_1_‐antitrypsin: α_1_‐antitrypsin polymers, the M* intermediate and a residual conformer that is most likely native monomer. Using MG132, the M* population was found to be unaffected by the ERAD pathway, indicating that this molecular state is either not recognised as aberrant or assimilated sufficiently rapidly into polymers to evade degradation. In contrast, the formation of M* was suppressed almost completely by c716, resulting in an increase in native monomer and inhibition of both soluble and insoluble polymer formation. Thus, both soluble and insoluble species have a common origin and are not formed by alternative pathways. The compound also restored the secretion profile of Z α_1_‐antitrypsin to that of the wild‐type protein [19, 34], demonstrating that M* is a key point in the process of Z α_1_‐antitrypsin folding, degradation and polymerisation within the ER of cells.

In summary, our data allow us to propose a model for the handling of Z α_1_‐antitrypsin within the ER. The nascent α_1_‐antitrypsin polypeptide folds via M* to native monomer to become incorporated into a polymer in the soluble fraction of the cell. This polymer can in turn become insoluble through mechanisms that have yet to be elucidated, can be secreted or be degraded. As these secreted polymers are a product of the intracellular processes of expression, M* formation, oligomerisation, soluble–insoluble partition and secretion, they potentially represent a useful reporter of intrahepatic polymerisation for polymer‐blocking therapies in individuals with α_1_‐antitrypsin deficiency.

## Materials and methods

### Inducible cell lines and iPSC‐derived hepatocytes

Chinese hamster ovary (CHO K1) cell lines expressing M or Z α_1_‐antitrypsin under the tetracycline‐inducible promoter [19] were maintained in Dulbecco's modified Eagle's medium (DMEM) supplemented with 10% v/v tetracycline‐free FBS (Takara Bio, Saint‐Germain‐en‐Layne, France), 1% w/v penicillin/streptomycin, 200 μg·mL^−1^ geneticin and 500 μg·mL^−1^ hygromycin B (both selective antibiotics from Invitrogen, Carlsbad, CA, USA) at 37 ºC and 5% v/v CO_2_. Before each experiment, cells were seeded at a density of 16.3 × 10^4^ cells·cm^−2^ and induced to express α_1_‐antitrypsin with 0.5 μg·mL^−1^ doxycycline for 48 h.

iPSC‐derived hepatocytes and supplements for cell media were supplied by DefiniGEN (DefiniGEN Ltd, Cambridge, UK). Cells were revived and maintained as advised by the supplier. Cells were differentiated in mature hepatocytes after 10 days of culture in hypoxic conditions (5% v/v CO_2_, 5% v/v O_2_, 90% v/v N_2_) in their recommended supplemented medium.

### Cell lysis, polymer extraction and immunoprecipitation

Both CHO K1 cells and iPSC‐derived hepatocytes were lysed at a concentration of 2.5 × 10^6^ cells·mL^−1^ in 1% v/v NP‐40 buffer (10 mm Tris, pH 7.4, 300 mm NaCl, 1% v/v NP‐40) supplemented with protease inhibitors (Roche Ltd, Hertfordshire, UK). Cell lysates were collected and mixed for 30 min at 4 ºC on a rotator mixer. 1% v/v NP‐40‐insoluble and NP‐40‐soluble fractions were separated by centrifugation at 16 000 ***g*** for 15 min at 4 ºC. The supernatant was collected (1% v/v NP‐40‐soluble fraction), while the pellet (1% v/v NP‐40‐insoluble fraction) was washed twice in 1% v/v NP‐40 buffer and mechanically resuspended in an equal volume of 1% v/v NP‐40 buffer supplemented with protease inhibitors. The 1% v/v NP‐40‐insoluble fraction was finally solubilised by sonication at 1.15 KHz (5 µm amplitude) for 15 s at RT (Soniprep 150; MSE Ltd, London, UK).

For analysis in nondenaturing PAGE, total α_1_‐antitrypsin from cell lysates and culture medium samples was concentrated using ConA‐conjugated agarose beads (Sigma‐Aldrich Co, Dorset, UK). Samples were diluted 1 : 2 with binding buffer (20 mm Tris, pH 7.0, 0.5 M NaCl) and incubated overnight on the rotator at 4 ºC. Elution was performed by incubating the beads with 1 M methyl‐α‐d‐mannopyranoside at 37 ºC for 2 h. The eluates were then resolved on 3–8% w/v nondenaturing PAGE (Bio‐Rad Laboratories Ltd, Hertfordshire, UK) and immunoblotted for total α_1_‐antitrypsin.

### Immunoprecipitation

For IP, cell lysates or culture media were mixed on the rotator with 1 µg of purified antipolymer 2C1 mAb [28] for 1 h at 4 ºC. Recombinant protein G agarose beads (Thermo Fisher, Loughborough, UK) were then added and the sample incubated on the rotator overnight at 4 ºC. The supernatant was collected and subjected to a further two rounds of IP with the 2C1 mAb, and the residual monomeric species of α_1_‐antitrypsin were immunoprecipitated with the antitotal α_1_‐antitrypsin 3C11 mAb [55]. For the experiment shown in Fig. 5B, the culture media and cell lysate samples were sequentially immunoprecipitated four times with the antipolymer 2C1 mAb, to ensure that all the polymers had been captured, once with anti‐M* intermediate 5E3 mAb [32] and finally with antitotal α_1_‐antitrypsin 3C11 mAb. At the end of each IP step, beads were collected, washed three times with 1% v/v NP‐40 buffer and once with 10 mm Tris, pH 7.4, and eluted in loading buffer (New England Biolabs Ltd, Hertfordshire, UK) in reducing conditions at 93 ºC for 5 min. The eluate was then resolved on 4–12% w/v acrylamide SDS/PAGE (Bio‐Rad Laboratories Ltd) followed by either immunoblotting with polyclonal rabbit anti‐α_1_‐antitrypsin (Dako, Agilent, CA, USA) or autoradiography, as detailed in figure legends.

Cells subjected to treatment with the experimental compound were incubated with 10 μm of the small molecule during the induction with doxycycline. After 48‐h induction, cells were washed in prewarmed PBS and incubated in Opti‐MEM (Thermo Fisher Scientific Ltd) for 4 h at 37 ºC. The culture media containing the experimental compound were changed every 48 h.

### Secretion assay and sandwich ELISA

After 48‐h induction with 0.5 µg·mL^−1^ doxycycline, CHO K1 cells were washed twice in prewarmed PBS and then incubated at 37 ºC with Opti‐MEM (Gibco, Thermo Fisher Scientific Ltd, Loughborough, UK). After a 12‐h incubation, culture medium was collected, centrifuged at 300 ***g*** for 5 min and 4 ºC, transferred into a clean tube and subjected to IP or concentration by ConA‐agarose resin. The serum samples from patients were analysed by sandwich ELISA as previously described [28].

### Metabolic labelling and pulse chase

Chinese hamster ovary K1 cells were labelled after 48‐h induction with 0.5 µg·mL^−1^ doxycycline and iPSC‐derived hepatocytes after differentiation to the hepatocyte stage. Cells were pulsed (0.45 MBq/10^6^ cells) for 10 min with ^35^S Cys/Met (EasyTag™ Express Protein Labelling; Perkin Elmer, Beaconsfield, UK) in DMEM without Cys/Met and then chased in normal culture medium for 0, 0.5, 1, 2, 4, 8 and 12 h. Long metabolic labelling (0.9 MBq/10^6^ cells) was performed with ^35^S Cys/Met for 12 h in the presence of cold methionine and cysteine. Cells were then cultured for 12 h in normal medium to promote the incorporation of all the radioactive monomeric α_1_‐antitrypsin into polymers. After the pulse, cells were chased at 0, 3, 12, 24, 36, 48, 60 and 72 h for CHO K1 cells and up to 108 h for iPSC‐derived hepatocytes. Radiolabelled α_1_‐antitrypsin was isolated by IP and resolved by SDS/PAGE followed by autoradiography. Densitometric analysis of α_1_‐antitrypsin bands was performed with image studio lite (LI‐COR Biosciences, Lincoln, Nebraska, USA). Statistical analysis was performed using the graphpad prism program (GraphPad Software, La Jolla, CA, USA).

### SDS/PAGE, native PAGE and immunoblot

Material obtained from IP or ConA‐agarose concentration as described in the previous section was resolved either by SDS/PAGE or by nondenaturing PAGE on pre‐cast NuPAGE™ 4–12% w/v acrylamide Bis/Tris Protein Gels and NativePAGE™ 3–12% w/v acrylamide Bis/Tris Protein Gels (Bio‐Rad Laboratories Ltd), respectively. Samples were then transferred to LF‐PVDF membranes (Millipore Ltd, Hertfordshire, UK). Membranes were saturated in 5% w/v low‐fat milk (Cell Signaling Technology, Danvers, MA, USA) (New England Biolabs Ltd) in PBS‐0.1% v/v Tween, probed with the indicated primary antibodies and horseradish peroxidase (HRP)‐conjugated secondary antibodies (Santa Cruz Biotechnology, Dallas, USA) and revealed by ECL (Clarity; Bio‐Rad Laboratories Ltd). Western blot images were acquired with the Image Quant Las400 (GE Healthcare Life Sciences, CA, USA) and analysed with image studio lite software (LI‐COR Biosciences, Cambridge, UK). Statistical analysis was performed using the graphpad prism program.

### Human plasma samples

All human biological samples were obtained with informed consent under an IRB/EC protocol approved by NRES Committee London‐Hampstead (REC Ref. 13/LO/1085, IRAS Project ID 130158, Study Title: Targeting Dysfunctional Mechanisms in α_1_‐antitrypsin deficiency).

## Conflict of interest

David Lomas is an inventor on patent PCT/GB2019/051761 that includes the small molecule inhibitor of polymerisation c716. The intellectual property has been transferred from GlaxoSmithKline to UCL Business who have licensed it to a third party.

## Author contributions

RR, ACP, JAI and DAL designed research; RR, NH‐C and JAI analysed data; RR, NH‐C and MR performed the research; and RR, AF, EM, JAI and DAL wrote the paper.

## Supporting information

**Fig S1.** Plasma samples from Fig. 3B were pulled down with concanavalinA (ConA), eluted and resolved on 4‐12% w/v acrylamide PAGE. Proteins were then transferred to a PVDF membrane and detected with 2C1 mAb. Densitometric analysis of the signals was performed with ImageStudio software and analysed with Graphpad Prism software. Graph shows mean ± standard error of the mean (±SEM, n=2) (One‐way ANOVA, Bonferroni multiple comparisons test, df=4).
**Fig S2.** CHO cells expressing Z a1‐antitrypsin were induced with 0.5 μg/mL doxycycline and treated with either the polymerisation inhibitor or 0.1% v/v DMSO for 48 h. 1 h before the beginning of the secretion assay, cells were treated with 80µM MG132, washed and incubated in OptiMEM for 4 h at 37ºC. The second, third and fourth IP with 2C1 mAb for 1% v/v NP‐40 soluble, insoluble and extracellular fractions were resolved after elution, on 4%‐12% w/v acrylamide SDS‐PAGE followed by immunoblotting for total a1‐antitrypsin. No 2C1 positive signal was detectable in the intracellular fraction after the third IP. One set of IP with 2C1 mAb was sufficient to deplete all the polymeric component in the EC.
