# Supplementary material for: The molecular species responsible for α1‐antitrypsin deficiency are suppressed by a small molecule chaperone
Source: FEBS J. 2020 Nov 11;288(7):2222–37. doi: 10.1111/febs.15597 (PMC8436759; doi:10.1111/febs.15597)
Supplement: Supplementary file 1 — Fig S1. Plasma samples from Fig. 3B were pulled down with concanavalinA (ConA), eluted and resolved on 4‐12% w/v acrylamide PAGE. Proteins were then transferred to a PVDF membrane and detected with 2C1 mAb. Densitometric analysis of the signals was performed with ImageStudio software and analysed with Graphpad Prism software. Graph shows mean ± standard error of the mean (±SEM, n=2) (One‐way ANOVA, Bonferroni multiple comparisons test, df=4). Fig S2. CHO cells expressing Z a1‐antitrypsin were induced with 0.5 μg/mL doxycycline and treated with either the polymerisation inhibitor or 0.1% v/v DMSO for 48 h. 1 h before the beginning of the secretion assay, cells were treated with 80µM MG132, washed and incubated in OptiMEM for 4 h at 37ºC. The second, third and fourth IP with 2C1 mAb for 1% v/v NP‐40 soluble, insoluble and extracellular fractions were resolved after elution, on 4%‐12% w/v acrylamide SDS‐PAGE followed by immunoblotting for total a1‐antitrypsin. No 2C1 positive signal was detectable in the intracellular fraction after the third IP. One set of IP with 2C1 mAb was sufficient to deplete all the polymeric component in the EC. [file FEBS-288-2222-s001.zip › febs15597-sup-0001-FigS1-S2.pdf]

FEBS PRESS
science publishing by scientists

WILEY

The FEBS Journal

www.febsjournal.org

The molecular species responsible for α1-antitrypsin deficiency are suppressed by a small molecule chaperone

Riccardo Ronzoni, Nina Heyer-Chauhan, Annamaria Fra, Andrew C. Pearce, Martin Rüdiger, Elena Miranda, James A. Irving and David A. Lomas

DOI: 10.1111/febs.15597

Fig.S1

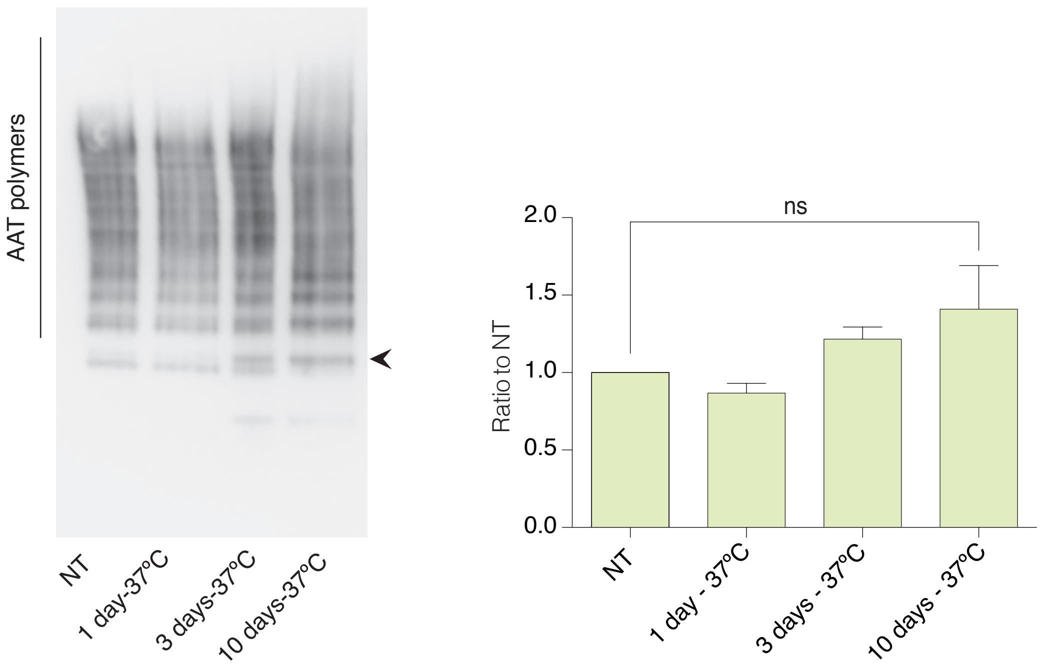
AAT polymers
NT
1 day-37ºC
3 days-37ºC
10 days-37ºC
ns
2.0
1.5
1.0
0.5
0.0
Ratio to NT
NT
1 day - 37ºC
3 days - 37ºC
10 days - 37ºC

Fig.S2

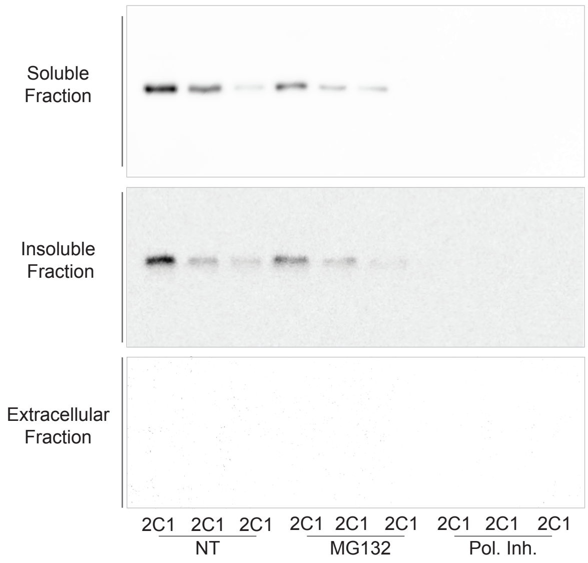
Soluble Fraction
Insoluble Fraction
Extracellular Fraction
2C1 2C1 2C1 2C1 2C1 2C1 2C1 2C1 2C1
NT
MG132
Pol. Inh.
